# Renin–Angiotensin–Aldosterone Inhibitors and COVID-19 Infection

**DOI:** 10.1007/s11906-022-01207-3

**Published:** 2022-06-18

**Authors:** Vasiliki Tsampasian, Natasha Corballis, Vassilios S. Vassiliou

**Affiliations:** 1 Norwich Medical School, University of East Anglia, Norwich, UK; 2 Norfolk and Norwich University Hospital, Norwich, UK

**Keywords:** Severe acute respiratory syndrome coronavirus 2 (SARS-CoV-2), Coronavirus disease 2019 (COVID-19), Renin–angiotensin–aldosterone system (RAAS), Angiotensin-converting enzyme 2 (ACE2)

## Abstract

**Purpose of Review:**

This review summarises the literature data and provides an overview of the role and impact of the use of renin–angiotensin–aldosterone system (RAAS) inhibitors in patients with coronavirus disease 2019 (COVID-19) infection.

**Recent Findings:**

The angiotensin-converting enzyme 2 (ACE2) has a key role in the regulation of the RAAS pathway, downregulating angiotensin II and attenuating inflammation, vasoconstriction and oxidative stress. Additionally, it plays an instrumental part in COVID-19 infection as it facilitates the cell entry of the severe acute respiratory syndrome coronavirus 2 (SARS-CoV-2) and enables its replication. The use and role of RAAS inhibitors therefore during the COVID-19 pandemic have been intensively investigated.

**Summary:**

Although it was initially assumed that RAAS inhibitors may relate to worse clinical outcomes and severe disease, data from large studies and meta-analyses demonstrated that they do not have an adverse impact on clinical outcomes or prognosis. On the contrary, some experimental and retrospective observational cohort studies showed a potential protective mechanism, although this effect remains to be seen in large clinical trials.

## Introduction

The renin–angiotensin–aldosterone system (RAAS) has a crucial role in the regulation of sympathetic system and tone and the control of the blood pressure, vascular tone and electrolyte balance. The RAAS inhibitors are commonly used in clinical practice as they are beneficial in the management of a range of cardiovascular diseases, such as hypertension, heart failure, myocardial infarction, with not only symptomatic but also prognostic benefit [1].

Since the beginning of the coronavirus pandemic, however, the medications involved in the RAAS came to the spotlight and attracted a lot of research interest. That is because coronaviruses, including the severe acute respiratory syndrome coronavirus (SARS-CoV), the Middle East Respiratory Syndrome (MERS-CoV) and the severe acute respiratory syndrome coronavirus 2 (SARS-CoV-2), use the angiotensin-converting enzyme 2 (ACE2) as their receptor in order to achieve their entrance in the host cell and trigger an immune reaction [2]. The first observational studies of the current pandemic showed a negative prognostic correlation between severe coronavirus disease 2019 (COVID-19) infection and a range of cardiovascular diseases in which RAAS inhibitors are commonly used [3–5]. Subsequently, hypotheses emerged that the use of medications that interfere with the RAAS axis have an implication on the morbidity, mortality and disease severity by increasing the circulating levels of ACE2 [6, 7]. Conflicting evidence from early observational studies contributed to the confusion of the general population and the scientific community as to whether the clinical course of the COVID-19 infection is truly affected by the potential chronic upregulation of the ACE2 receptors and how the administration of the RAAS inhibitors may impact and alter the disease process.

The aim of this review is to provide a comprehensive critical evaluation of the literature evidence on the role of renin–angiotensin–aldosterone inhibitors in COVID-19 infection and the prognostic and clinical implications of these medications in the disease process induced by the SARS-CoV-2 virus.

## COVID-19 and the RAAS Axis

**Fig. 1:**
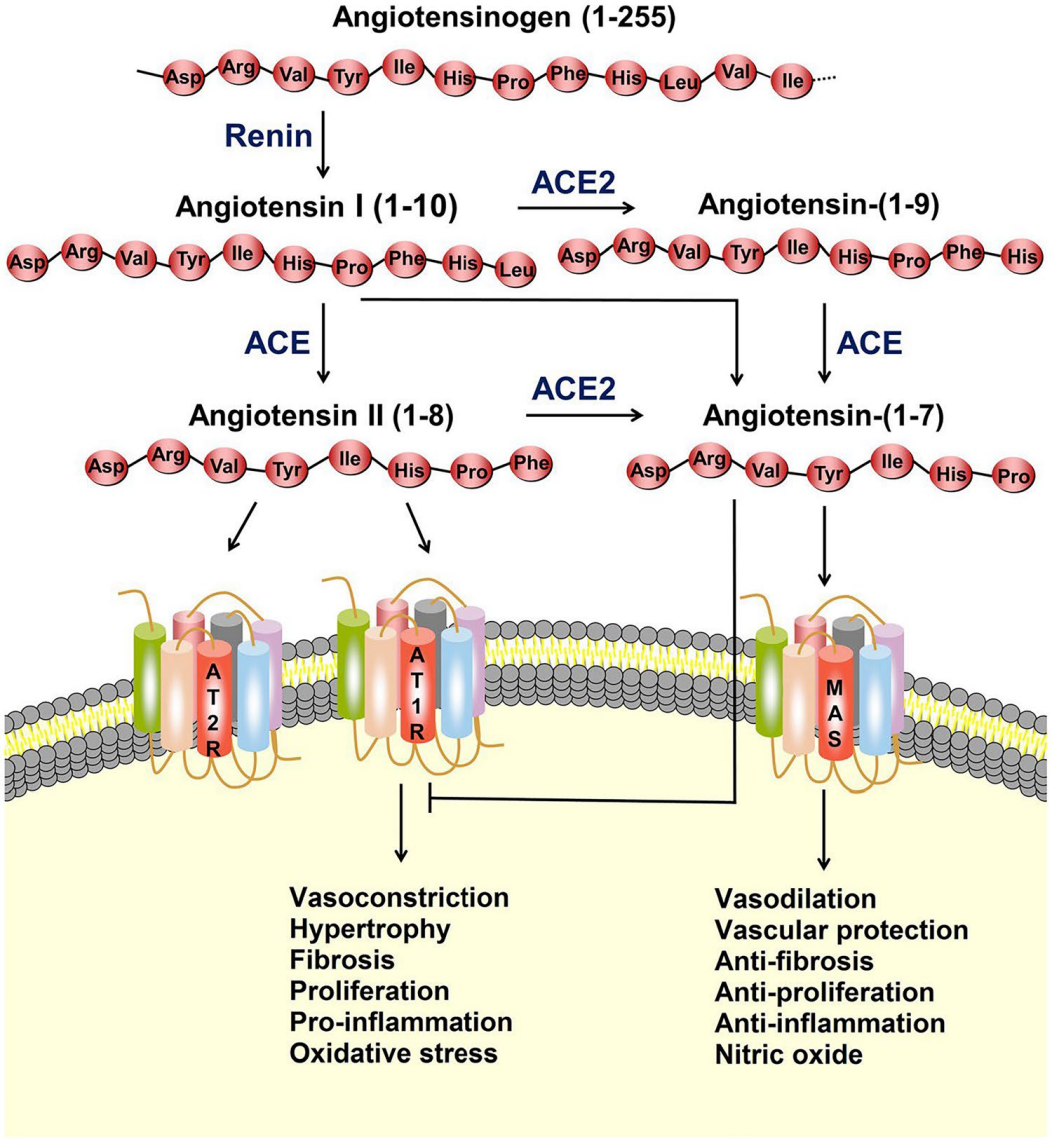
The renin–angiotensin system (RAS) and ACE2/angiotensin- [1–7] /MAS axis. The protease renin converts angiotensinogen to Ang-I, which is subsequently converted to Ang-II by angiotensin-converting enzyme (ACE). Ang-II can bind to the angiotensin type 1 receptor (AT1R) to exert actions, such as vasoconstriction, hypertrophy, fibrosis, proliferation, inflammation and oxidative stress. ACE2 can covert Ang-I and Ang-II to angiotensin- [1–7]. Angiotensin- [1–7] binds to the MAS receptor to exert actions of vasodilation, vascular protection, anti-fibrosis, anti-proliferation and anti-inflammation. Ang-II can also bind to the angiotensin type 2 receptor (AT2R) to counteract the aforementioned effects mediated by AT1R [11•]. Reproduced with permission from Ni et al. [11•] under a Creative Commons Attribution 4.0 International License

ACE2 is a crucial enzyme of the RAAS pathway, present in the epithelial cells of multiple organs, including the lungs, heart, blood vessels, gastrointestinal tract and kidneys [2]. It is a key counter-regulatory component of angiotensin metabolism that was identified approximately twenty years ago [8, 9]. One of its main metabolic actions includes the breakdown of angiotensin II to ang- [1–7] (Fig. 1) [10, 11•]. Angiotensin II has a major impact on blood pressure and cardiac remodelling [12]. Binding to the angiotensin II type 1 receptor (AT1R) to initiate its actions, angiotensin II is a potent vasoconstrictor with a deleterious effect upon tissues of various organs as it promotes inflammation, fibrosis and oxidative stress [12, 13]. By breaking down angiotensin II, ACE2 has therefore a protective role of paramount importance in cardiovascular homeostasis. Ang- [1–7] acts through the Mas receptor and promotes vasodilation, sodium and water excretion as well as attenuation of inflammation and fibrosis, counter-acting in this way the effects of angiotensin II signalling [6].

**Fig. 2:**
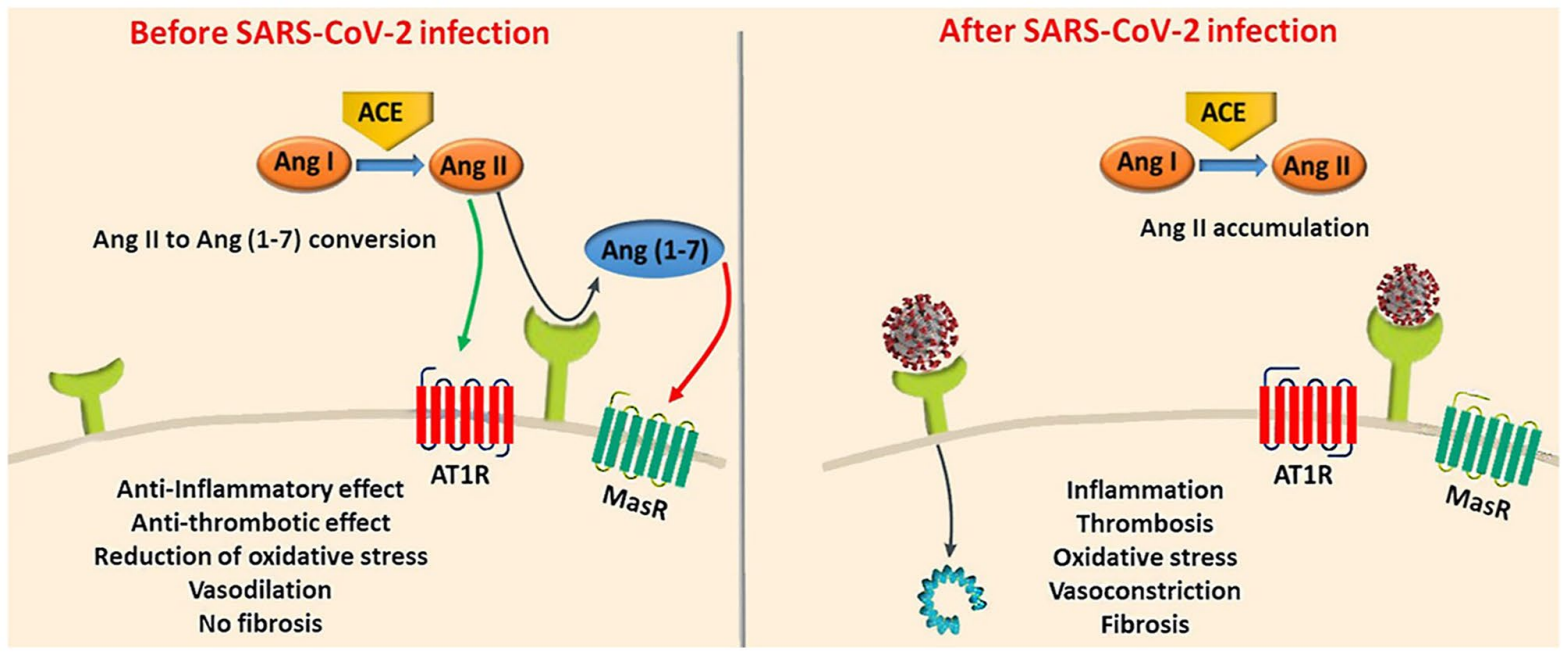
In healthy individuals, angiotensin II is converted into angiotensin [1–7] via ACE2. However, in COVID-19, ACE2 may be dysfunctional due to the binding of SARS-CoV-2, which can affect the conversion of angiotensin II to angiotensin [1–7]. This results in the accumulation of angiotensin II in the infected person and induces proinflammatory, prothrombotic, fibrotic and vasoconstrictive downstream effects. In the presence of CVD, however, the RAAS could be impaired. Consequently, upon infection with SARS-CoV-2, more angiotensin II could accumulate resulting in serious cardiovascular complications [16]. Reproduced with permission from Augustine et al. [16] under a Creative Commons Attribution 4.0 International License

Recently, it became evident that ACE2 also has a central role in the pathogenesis of COVID-19 disease. COVID-19 is caused by the SARS-CoV-2, the latest of the coronaviruses that emerged in Wuhan in December 2019 [14]. Similarly with the other coronaviruses, SARS-CoV-2 uses surface spike (S) proteins to enter the host cell. The entry of the virus into the host cell is facilitated through the attachment of the S protein to the angiotensin-converting enzyme 2 (ACE2) and the proteolytic cleavage of the S protein by the transmembrane serine protease 2 (TMPRSS2) (Fig. 2) [6, 15, 16]. In fact, the novel SARS-CoV-2 has been found to form a much stronger attachment with ACE2 compared to the SARS-CoV [17, 18].

Although COVID-19 is primarily a respiratory disease, it is well known that its course may have a detrimental effect on the cardiovascular system with resultant acute or sub-acute myocardial injury and/or inflammation [19, 20]. Given that ACE2, one of the key molecules in cardiovascular physiology and counter-regulator of the RAAS axis, is also an important host receptor that the virus uses to enter the cells and trigger the infection, it is obvious that it can become an easy—and, arguably, a rational—research target to focus on.

## ACE2 and SARS-CoV

ACE and ACE2 enzymes share similar structures with ACE2 being homologous to one of the active sites of ACE [21]. The two enzymes, however, do have differences in their structure, and they share 40% overall identity [21]. Angiotensin-converting enzyme inhibitors (ACEi) or angiotensin receptor blockers (ARBs) do not bind on ACE2, and therefore, they do not impact ACE2 directly [13, 21]. However, ACE2 gene and protein expression have been shown to be increased by RAAS inhibitors in published data from animal studies [22–24]. Its expression has also been shown to be dysregulated in cardiac diseases of different aetiologies, such as pressure overload conditions from valvular heart disease and cardiomyopathies [25, 26]. Concerns were therefore expressed, and the hypothesis was generated that patients with underlying chronic cardiovascular comorbidities regularly taking RAS inhibitors would be at higher risk of developing severe infection, as upregulation of ACE2 receptors may translate in high numbers of available entry points that the virus can ‘use’ to invade the host cells, replicate itself and trigger a potentially severe inflammatory reaction [6, 7].

The fact that coronaviruses dysregulate the RAAS axis has been known for almost two decades. With the emergence of SARS coronavirus in 2003, ACE2 was identified as the key receptor that facilitates the entry of the virus into the cells [27••]. As discussed already, the initial concerns about the use of ACEi and ARBs were based on the fact that SARS-CoV-2 cell entry is mediated by the ACE2 receptor [12]. With studies showing that RAAS inhibitors can increase the ACE2 expression, it was hypothesised that patients infected with SARS coronavirus had increased risk of severe disease, as the high number of ACE2 receptors would allow the virus to enter the host cells through many entry points leading to extensive viral replication, cellular death and triggering in this way severe organ injury [12, 13, 28]. However, in contrast to the hypothesis above, experimental studies supported the theory that ACE2 has a crucial protective role in the SARS-CoV-mediated infection [27••]. While ACE is the key enzyme that generates angiotensin II from angiotensin I, ACE2 inactivates and downregulates angiotensin II, which promotes acute and severe lung injury via the angiotensin II receptor type 1 (AT1R) [27••]. Subsequently, downregulation of ACE2 caused by the binding of the SARS-CoV spike protein to it may lead to an unheralded acute severe lung injury mediated by angiotensin II and its receptor AT1R [6, 27••]. This was confirmed by experimental studies using animal models, in which SARS-CoV-2-infected mice that suffered severe acute lung injury, a hallmark of the clinical presentation of COVID-19 infection, exhibited marked downregulation of the ACE2 protein expression and increased levels of angiotensin II [27••, 29]. In these models, the severe acute lung injury was mediated via angiotensin II and its receptor AT1R [27••, 29]. In the experimental study by Kuba et al., it was demonstrated that ARBs had a beneficial effect on SARS-Cov2-infected mice as they attenuated acute severe lung injury and pulmonary oedema by inhibiting the AT1R [27••].

## RAAS Inhibitors and COVID-19 Infection

Early observational studies made it clear that certain comorbidities are associated with higher risk of developing severe disease and/or death [3–5]. These included hypertension, heart failure, coronary heart disease, cerebrovascular disease and diabetes mellitus. In a retrospective study, Wu et al. found that patients with hypertension were 1.82 times more likely to develop ARDS than those without hypertension, while those with diabetes had 2.34 times higher risk to develop ARDS than those who did not have diabetes [3]. In another study that included more than a thousand patients, 23.7% of those who developed severe disease had pre-existing hypertension [5]. A further multi-centre cohort study revealed a strong association between hypertension, coronary heart disease and diabetes with in-hospital mortality from COVID-19 [4]. It has to be noted that none of the aforementioned studies assessed the direct impact of the commonly used medications for these co-morbidities, i.e. the ACEIs and ARBs, on COVID-19 severity and mortality. The research debate, however, over the actual clinical impact of the RAAS inhibitors on COVID-19 infection led to the conduction of several observational studies that evaluated the potential link of these medications with clinical outcomes and prognosis.

The initial results of these studies were as controversial as the two theories that supported them. Selçuk et al. in a study that included 113 hypertensive patients showed that the use of RAAS inhibitors was independently associated with higher in-hospital mortality [28]. Similarly, in a larger-scale observational study, Mehta et al. demonstrated that COVID-19-infected patients that were taking ACEI/ARBs had higher risk of hospital admission [30]. Subsequent large-scale studies, however, showed that there was no association between the use of these medications with the occurrence of a positive test or the clinical course and severity of the infection [31–33]. A few of them highlighted the fact that older age along with comorbidities, such as hypertension, may be important risk factors in prognosis and clinical outcomes of patients with COVID-19, but the use of ACEI/ARBs was not one of them [34, 35]. These findings were confirmed in a large observational study that included 8,910 patients from 169 hospitals in Asia, Europe and North America, which showed that underlying cardiovascular comorbidities, and not the use of ACEI or ARBs, were associated with a higher risk of severe disease and mortality from COVID-19 [36]. Despite the limitations of the retrospective observational nature of these studies, the undisputable findings were strongly supported by several scientific societies who urged clinicians and the public not to stop medications important for underlying chronic diseases over hypothetical concerns [37••, 38, 39].

The first randomised trials on this important topic soon came to validate the findings of the observational studies. The BRACE CORONA study included 659 patients with mild-to-moderate COVID-19 infection who were regularly taking ACEIs or ARBs. It supported the notion of the scientific societies by demonstrating that neither continuation nor discontinuation of these medications had a significant impact on the mortality or COVID-19 progression [40]. Three further smaller-scale randomised trials also demonstrated the safety of continuation of RAAS inhibitors in patients with COVID-19 infection [41–43]. A non-pre-specified interim analysis of 102 participants of the RASTAVI (Renin-Angiotensin System Blockade Benefits in Clinical Evolution and Ventricular Remodeling After Transcatheter Aortic Valve Implantation) trial showed that, even in this high-risk cohort of patients, RAAS inhibitors did not affect the impact or the severity of COVID-19 infection [44].

**Fig. 3:**
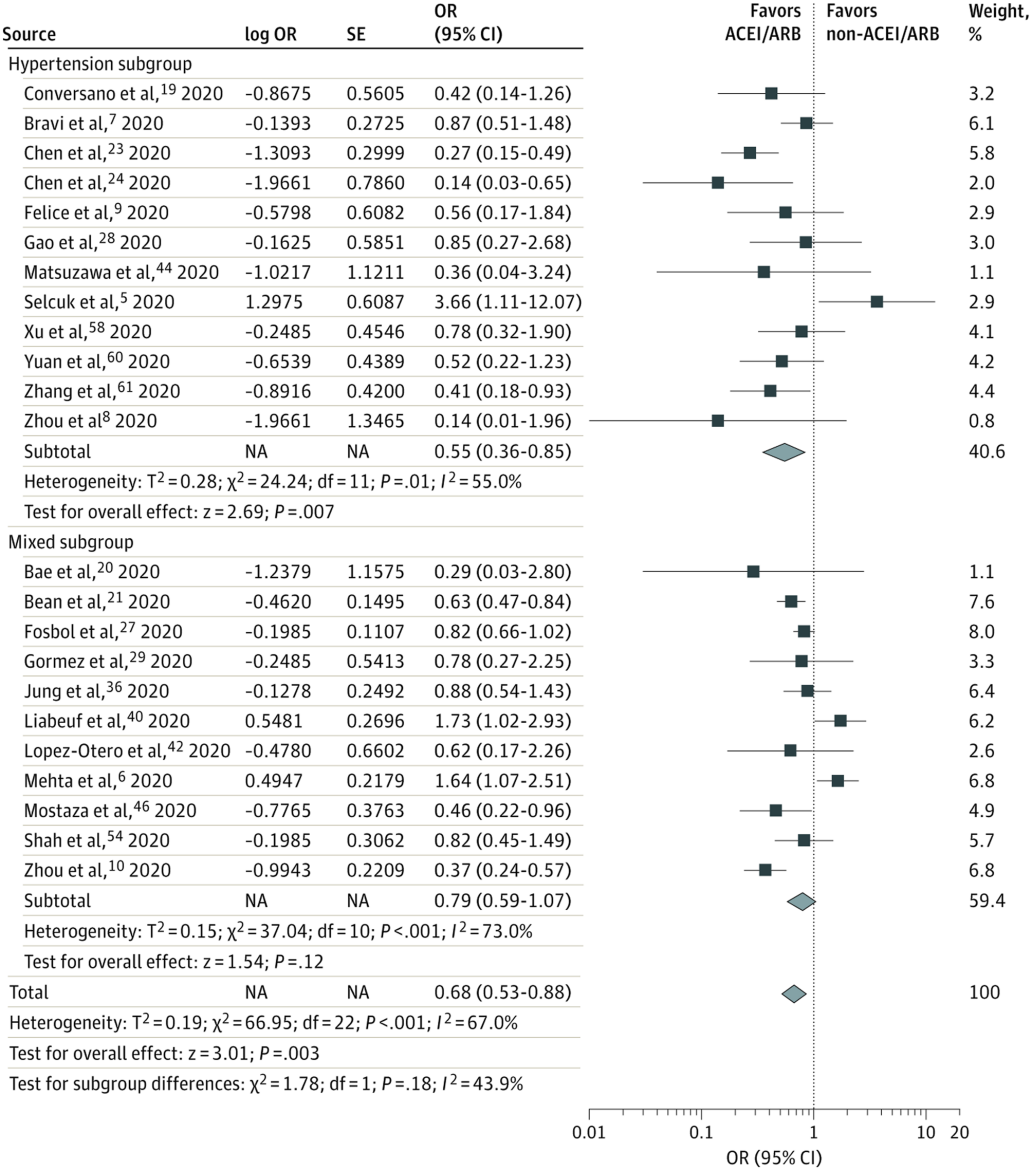
In the meta-analysis by Baral et al., subgroup analysis of adjusted mortality and severe adverse events among patients who did and did not receive angiotensin-converting enzyme inhibitor (ACEIs) or angiotensin receptor blocker (ARBs), showed that these medications have a protective effect on patients suffering with COVID-19 infection. A total of 11 studies included a mixed subgroup (sample population with multiple mixed comorbidities), and 12 studies included a hypertension subgroup (defined as a sample population with hypertension). Diamonds represent 95% confidence intervals for subtotal and total odds ratios (OR). Reproduced with permission from Baral et al. [53••] under a Creative Commons Attribution 4.0 International License

In a much evolving research landscape of COVID-19, the number and scale of the studies that appeared rose exponentially. The meta-analyses that surfaced reiterated that RAAS inhibition does not affect the clinical course or outcomes of COVID-19 disease [45, 46]. The emerging results though were much more favourable of the use of RAAS inhibitors, even demonstrating that they have a protective effect. In a multi-centre study that included 15,504 patients hospitalised with COVID-19 infection, Zhou et al. demonstrated that the use of ACEI/ARBs was associated with lower 28-day in-hospital mortality [47]. Likewise, a retrospective multi-centre study conducted by Yan et al., demonstrated that the use of RAAS inhibitors was associated with improved clinical outcomes in hospitalised patients with COVID-19 infection [48]. Further studies reinforced these findings by revealing lower rates of incidence of severe disease among the patients taking RAAS inhibitors [49–52]. In a meta-analysis consisted of a total population of 101,949 patients with COVID-19, Baral et al. confirmed that the use of ACEI or ARBs not only does not carry an increased risk of mortality or severe adverse events but also, quite the opposite, that it may have a protective effect (Fig. 3) [53••].

## RAAS Inhibitors as Therapeutic Strategy

Based on the hypothesis-generating experimental studies that showed the benefit of ARBs via the inhibition of AT1R, Duarte et al. conducted a parallel-group, randomised, open-label superiority trial that examined the use of telmisartan, a widely used ARB, in 158 patients with COVID-19 infection. Although the study was terminated early due to recruitment challenges, it showed that telmisartan reduced 30-day mortality and morbidity among hospitalised patients [54]. Interestingly, a similar effect was not shown in patients with mild COVID-19 infection managed in the outpatient setting [55]. In a randomised placebo-controlled trial that included 117 patients, the use of losartan did not have any impact on the rate of hospitalisations or on the individuals’ viral load [55]. Even though assessment was limited by a very low event rate in this trial, the results suggest that RAAS inhibition may become significant in cases of severe disease and unheralded inflammatory response and not in the first stages of viral entry and replication. Surprisingly, in a randomised controlled trial that included 205 hospitalised patients with acute lung injury, losartan did not have an impact on clinical outcomes and did not improve lung function [56]. Firm conclusions, however, cannot be drawn from these studies given the small number of participants, the different settings and the different pharmacological agents that were used.

Given that ACE2 counteracts the pro-inflammatory AT1R-mediated actions of angiotensin II, research studies have set to determine whether the use of recombinant human ACE2 (rhACE2) is of benefit in patients with COVID-19 infection and severe lung injury. The safety of rhACE2 had been tested before the COVID-19 in patients with acute respiratory distress syndrome (ARDS) and in healthy volunteers with encouraging results [57, 58]. In an experimental study by Monteil et al., it was shown that in engineered human organoids recombinant human-soluble ACE2 markedly reduced the SARS-CoV-2 load by a factor of 1000–5000 and directly neutralised the virus [59]. The results of the ongoing randomised controlled trial examining the use of rhACE2 in the management of patients with COVID-19 infection are greatly anticipated to contribute to our understanding on the role of ACE2 in the clinical course of this viral disease (*NCT04335136*).

In summary, despite the fact that the initial concerns and hypotheses that emerged during the COVID-19 pandemic resulted in patients worldwide stop taking their medication, the medical community quickly came together to assess the evidence and closely examine a potential risk–benefit imbalance. Starting from small observational studies, which subsequently led to large meta-analyses, it became clear that there was no sign of adverse prognosis directly relating to the RAAS inhibitors. In fact, the reason that many patients on RAAS inhibitors exhibited signs of severe infection or higher rates of mortality was because of the important pre-existing comorbidities and not the medications used to treat those, i.e. the RAAS inhibitors. Once the mortality and morbidity ratios were adjusted for these co-morbidities, it was clear that the RAAS inhibitors not only did not cause harm but, on the contrary, they likely had a protective effect [53••].

## Conclusions

ACE2 has established its crucial role not only in the regulation of the RAAS axis but also as a receptor of the SARS-CoV-2 in the host cells facilitating viral cell entry. Despite the initial theoretical concerns that RAAS inhibitors may be related to severe infection and adverse events, data from large studies and meta-analyses have demonstrated their use is safe in the patients suffering with COVID-19 infection, with evidence showing a beneficial effect. Although current studies so far have demonstrated that their commencement solely for protection against COVID-19 infection or its complications is not advantageous, there is strong evidence confirming that they are safe and should be continued by all individuals that have a clear indication to take them for an underlying cardiovascular disease. Further studies and randomised trials are warranted to examine the potential protective effect of these medications in severe COVID-19 disease and associated acute lung injury in patients without an underlying cardiovascular indication for them.
